# Black Clouds over Hospitals

**Published:** 1891-01-03

**Authors:** 


### BLACK CLOUDS OVER HOSPITALS.

If Zadkiel had cast the horoscope of the hospitals at the beginning of last year he would have announced "thunder-clouds, wars, prodigies, and portents." Never was there a year within our recollection when hospitals were so investigated, cross-examined, suspicioned, to coin a word, and criticised as they have been during the past twelve months. The Lords' Committee was welcomed by hospital managers, because they felt sure that it would prove a vindicator of their work, and give a fresh impulse to all their activities. It has not proved anything as yet, except its incapacity for distinguishing between essentials and accidentals, and its keen love of the minute female gossip which constitutes the staple of conversation at afternoon teas. Whether such an appetite for gossip is consistent with a Lords' Committee we must leave the lords themselves to decide. They have still much ground to cover, and we must hope that in their future proceedings a properly judicial temper will not be wanting. The London Hospital and its matron have been put into the fire; or shall we say into the lion's den? They have come out again, like certain famous patriots of old, with "not a bone broken, nor a hair of their head singed." It is passing strange, but a happy encouragement to well-doing, that, notwithstanding the gauntlet which the hospitals have had to run during the year, their public benefactions should have reached a higher level than they have ever attained before. The Hospital Sunday Fund amounted to nearly £42,000, and was more than £1,000 in excess of any previous series of collections. In the Saturday Fund the proportionate increase was decidedly greater still. In spite, however, of these encouraging facts, there was an excess of £75,000 in expenditure as compared with income in the Metropolitan hospitals alone. The total income of those hospitals from all sources was £396,789; the expenditure was £571,583. On behalf of the Sunday Fund, the usual annual meeting was held at the Mansion House, and Mr. Jonathan Hutchinson, F.R.S., President of the Royal College of Surgeons, delivered an address full of originality, power, and sound argument. That address was a complete vindication of the aims and work of the voluntary hospitals of Great Britain.

